# Supplementary material for: Single platinum atoms embedded in nanoporous cobalt selenide as electrocatalyst for accelerating hydrogen evolution reaction
Source: Nat Commun. 2019 Apr 15;10:1743. doi: 10.1038/s41467-019-09765-y (PMC6465355; doi:10.1038/s41467-019-09765-y)
Supplement: Supplementary file 1 — Supplementary Information [file 41467_2019_9765_MOESM1_ESM.pdf]

Supplementary Information

Single Platinum Atoms Embedded in Nanoporous Cobalt Selenide as Electrocatalyst for Accelerating Hydrogen Evolution Reaction

Jiang et al.

Supplementary Figures

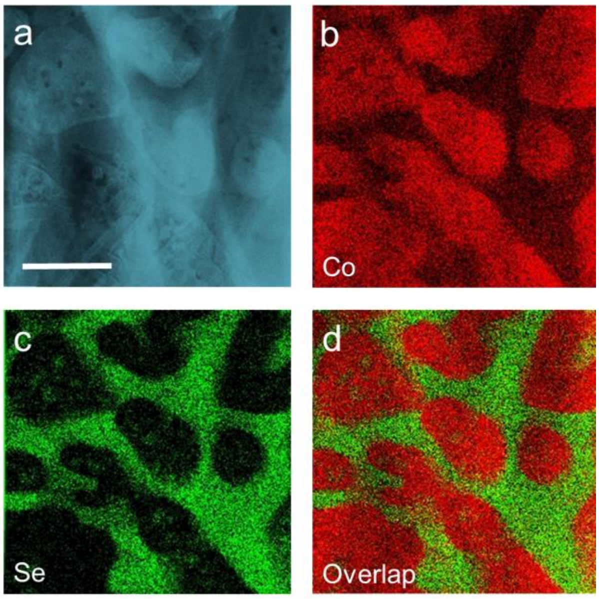

Supplementary Figure 1. HAADF-STEM characterizations of rapidly solidified $Co_{85}Se_{15}$ ribbon

(**a**) HAADF-STEM image of the rapidly solidified $Co_{85}Se_{15}$ ribbon. (**b-d**) STEM-EDS element mappings of $Co_{85}Se_{15}$ ribbon. Scale bar: 100 nm.

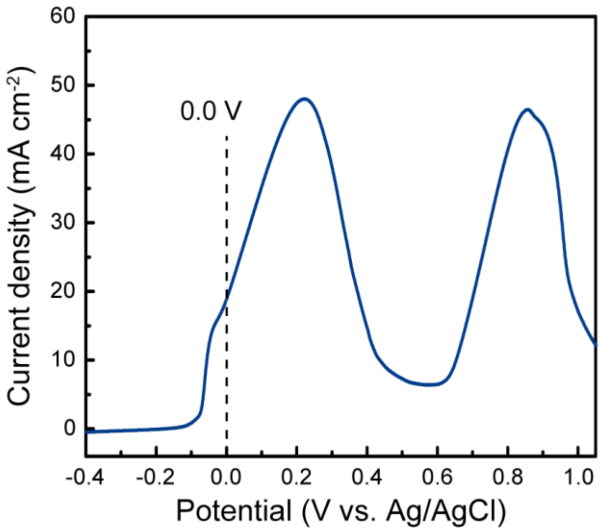

Supplementary Figure 2. The disparity in electrochemical stability

The linear sweep voltammetry curve of the $Co_{85}Se_{15}$ ribbon in 0.5 M H_2SO_4. There are two distinct peaks which are assigned to the oxidation region of the Co and $Co_{0.85}Se$ phases, respectively. The selective dissolution potential of the Co phase is -0.1 V vs. Ag/AgCl while it is 0.6 V for the dissolution of the $Co_{0.85}Se$ phase. Here we selected the corrosion potential of 0.0 V to selectively dissolve the Co phase, while the $Co_{0.85}Se$ phase is retained.

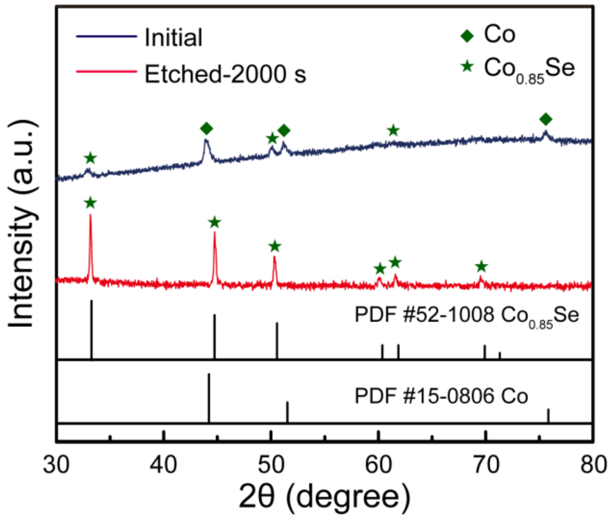

Supplementary Figure 3. XRD spectra of the initial $Co_{85}Se_{15}$ ribbons and np-$Co_{0.85}Se$ ribbons

The rapidly-solidified ribbons contain two crystalline phases: face-centered cubic Co and hexagonal $Co_{0.85}Se$. The Co phase was selectively dissolved from the two-phase ribbons in a H_2SO_4 solution.

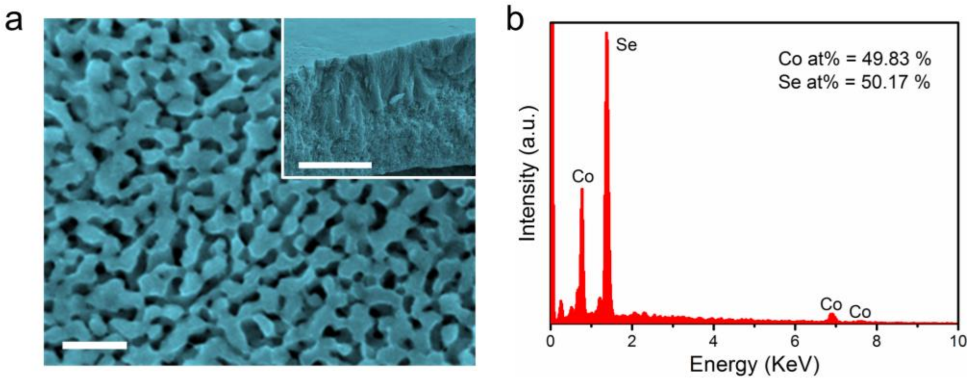

Supplementary Figure 4. Morphology characterizations of np-$Co_{0.85}$Se ribbon

(**a**) SEM image of np-$Co_{0.85}$Se ribbon. Inset shows the cross section of ribbon. (**b**) EDS spectrum, showing the compositions of the np-$Co_{0.85}$Se ribbon. Scale bar: (**a**) 200 nm, inset: 10 μm.

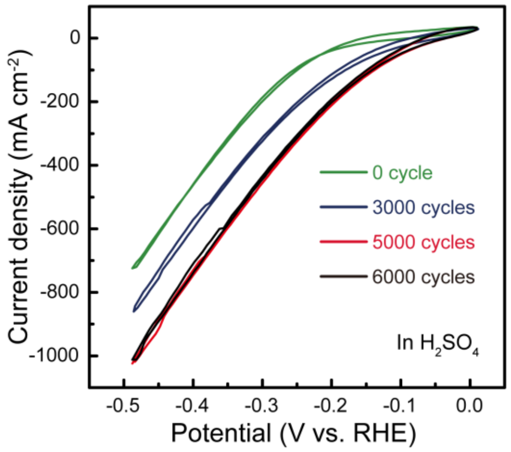

Supplementary Figure 5. Cyclic voltammetry for Pt deposition on the catalyst

Cyclic voltammetric curves of the np-$Co_{0.85}Se$ precursor sample after different potential cycles. These curves were taken with 50 mV s^{-1} in Ar-saturated 0.5 M H_2SO_4 at room temperature (without corrected for *iR* losses).

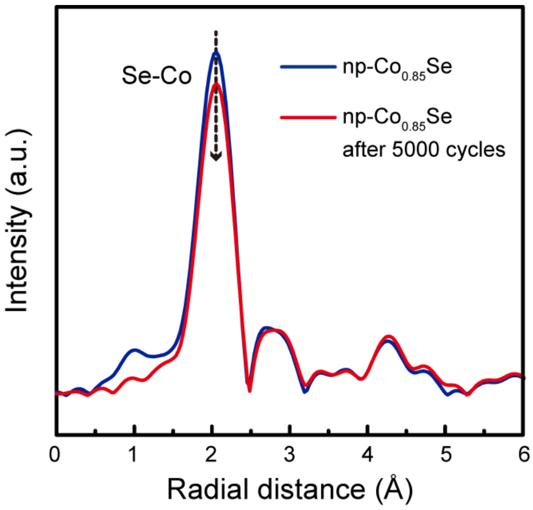

Supplementary Figure 6. FT-EXAFS spectra in real space at Se K-edge

Blue curve: np-$Co_{0.85}Se$. Red curve: np-$Co_{0.85}Se$ obtained after 5000 potential cycles using graphite sheet as counter electrode in a three-electrode cell containing 0.5 M H_2SO_4.

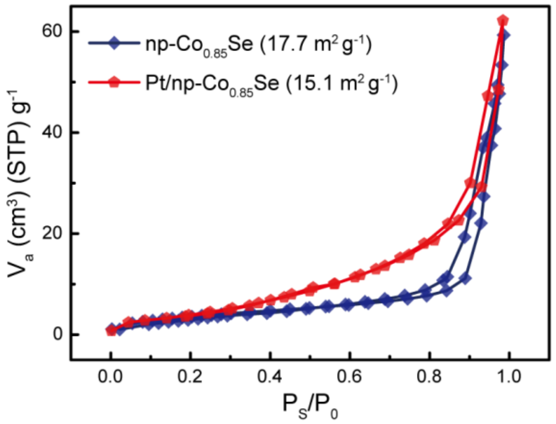

Supplementary Figure 7. BET surface area

N_2 adsorbing-desorbing isotherm curves of np-$Co_{0.85}Se$ and Pt/np-$Co_{0.85}Se$.

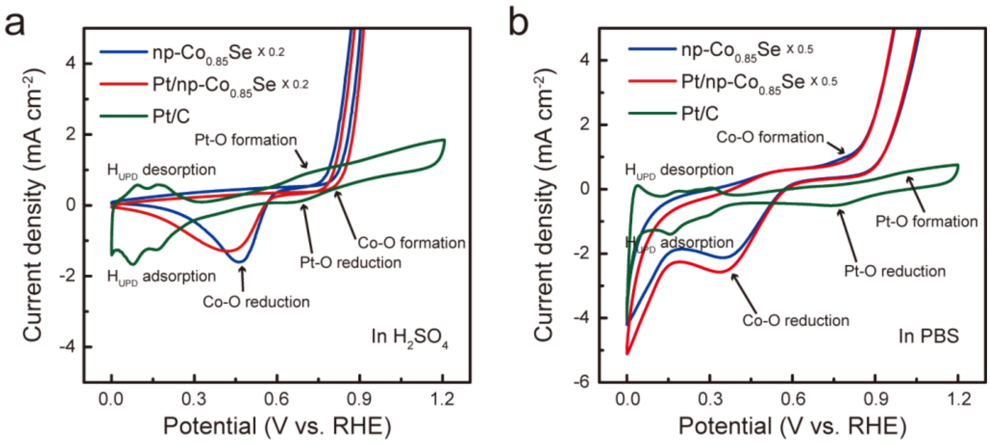

Supplementary Figure 8. CV measurements for Pt detection

Cyclic voltammograms (CVs) of np-$Co_{0.85}Se$, Pt/np-$Co_{0.85}Se$, and Pt/C. CV measurements were taken in N_2 purged (**a**) 0.5 M H_2SO_4 and (**b**) 1.0 M PBS with a scan rate of 50 mV s^{-1}.

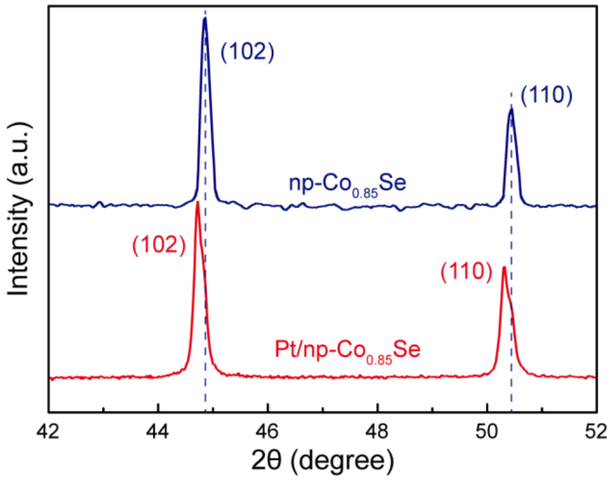

Supplementary Figure 9. XRD characterizations after single-atom Pt doping

XRD results at (102) and (110) peaks of np-$Co_{0.85}Se$ and Pt/np-$Co_{0.85}Se$.

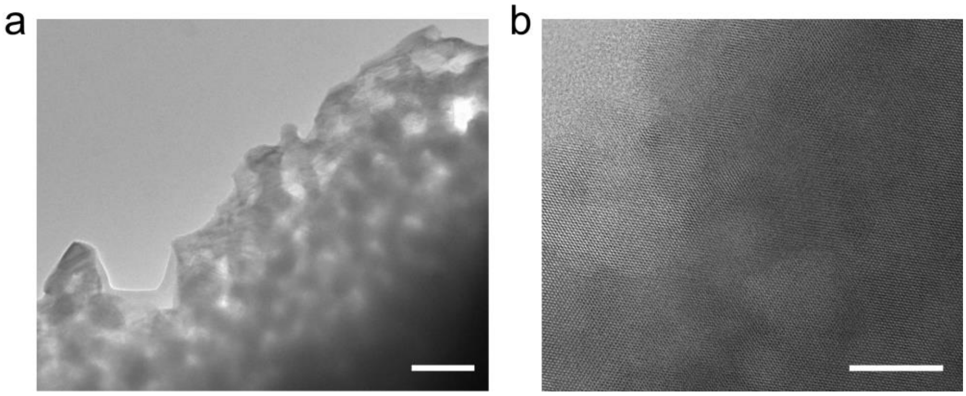

Supplementary Figure 10. TEM characterizations of Pt/np-$Co_{0.85}$Se

(**a**) TEM image of Pt/np-$Co_{0.85}$Se. (**b**) High-resolution TEM (HRTEM) image corresponding to (**a**). Scale bars: (**a**) 100 nm, (**b**) 10 nm.

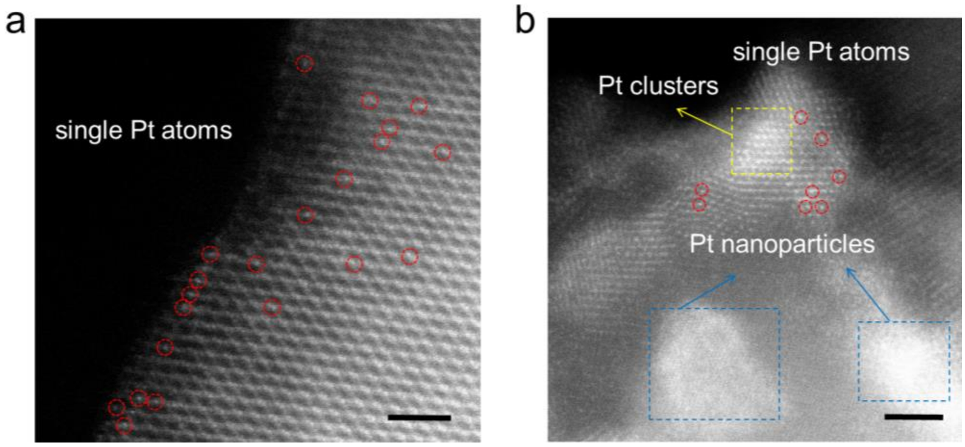

Supplementary Figure 11. HAADF-STEM characterizations

HAADF-STEM images of (**a**) Pt_S/np-$Co_{0.85}Se$ and (**b**) Pt_N/np-$Co_{0.85}Se$. Scale bars: (**a**) 1 nm, (**b**) 2 nm.

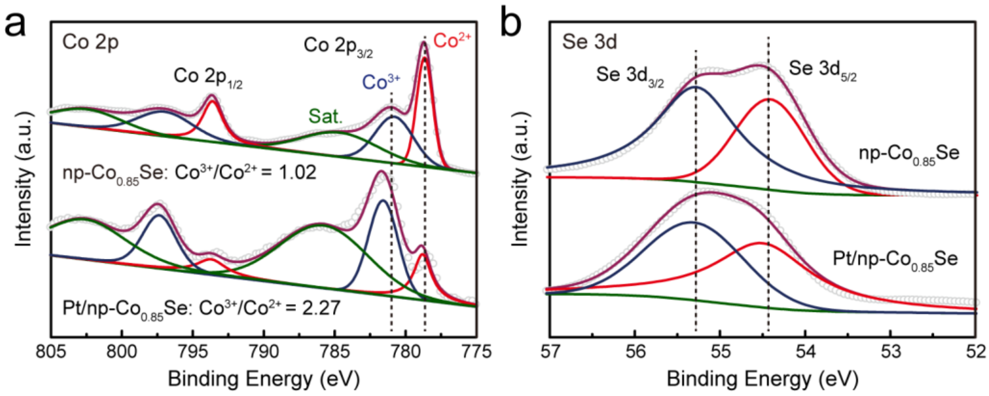

Supplementary Figure 12. XPS characterizations

XPS spectra of np-$Co_{0.85}Se$ and Pt/np-$Co_{0.85}Se$. (**a**) Co 2p, (**b**) Se 3d.

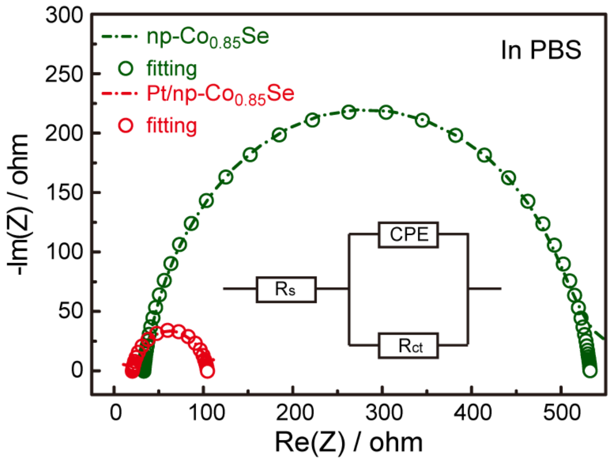

Supplementary Figure 13. Electrochemical impedance spectroscopy analyses

Nyquist plots of np-$Co_{0.85}Se$ and Pt/np-$Co_{0.85}Se$ at -50 mV vs. RHE. The inset is the equivalent circuit model that contains the electrolyte resistance (Rs), constant phase element (CPE) and charge-transfer resistance (RCT).

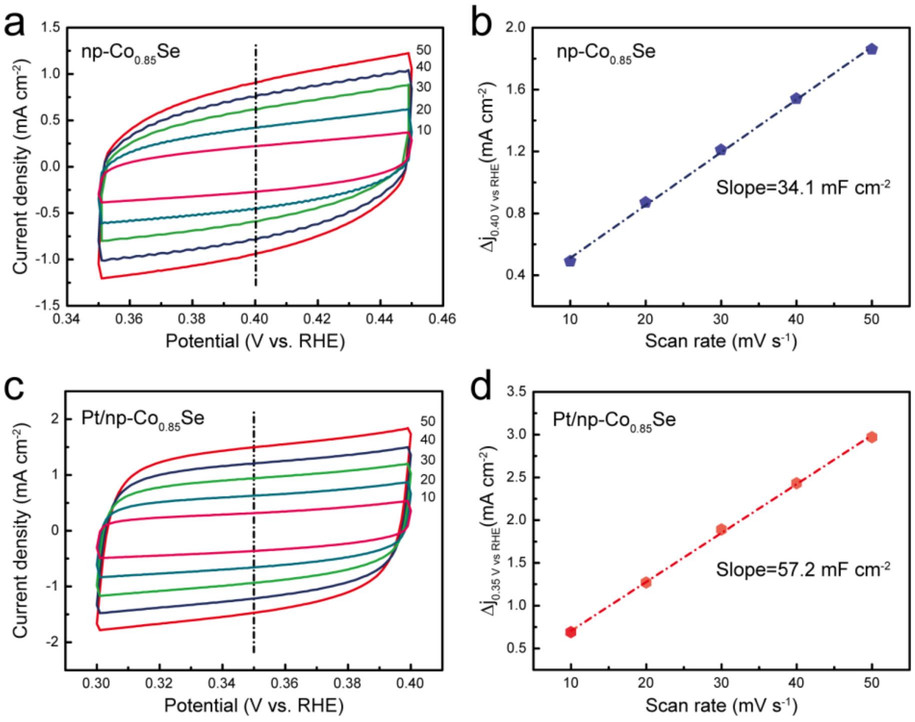

Supplementary Figure 14. Double-layer capacitance analyses

CVs of (**a**) np-$Co_{0.85}Se$ and (**c**) Pt/np-$Co_{0.85}Se$. These CVs were performed at various scan rates (10, 20, 30, 40, and 50 mV s^{-1}). (**b**, **d**) The plots of current densities against scan rates. Δj is the difference between anodic and cathodic current densities at a same potential (**b**: 0.40 V, **d**: 0.35 V).

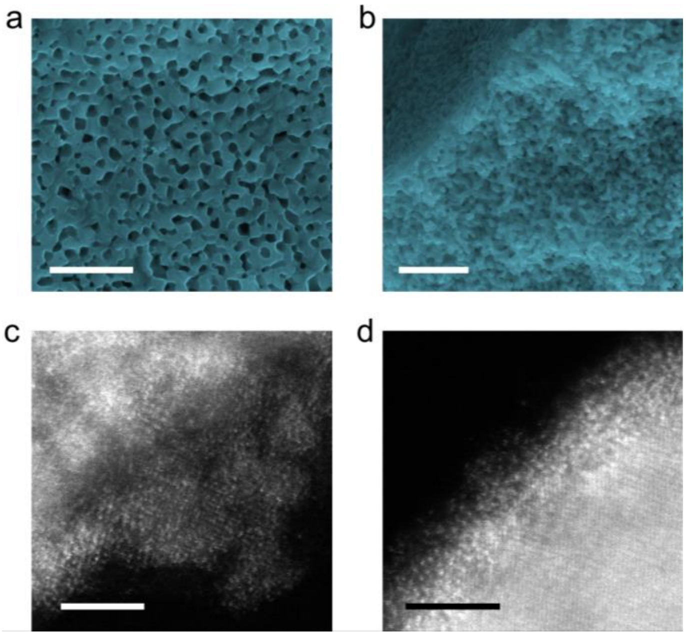

Supplementary Figure 15. Images after long-time operation

(**a**) SEM image, (**b**) cross-section SEM image and (**c**, **d**) HAADF-STEM images of Pt/np-$Co_{0.85}Se$ obtained after long-time operation in neutral solution. Scale bar: (**a**) 500 nm, (**b**) 1 μm, (**c**) 2 nm, (**d**) 2 nm.

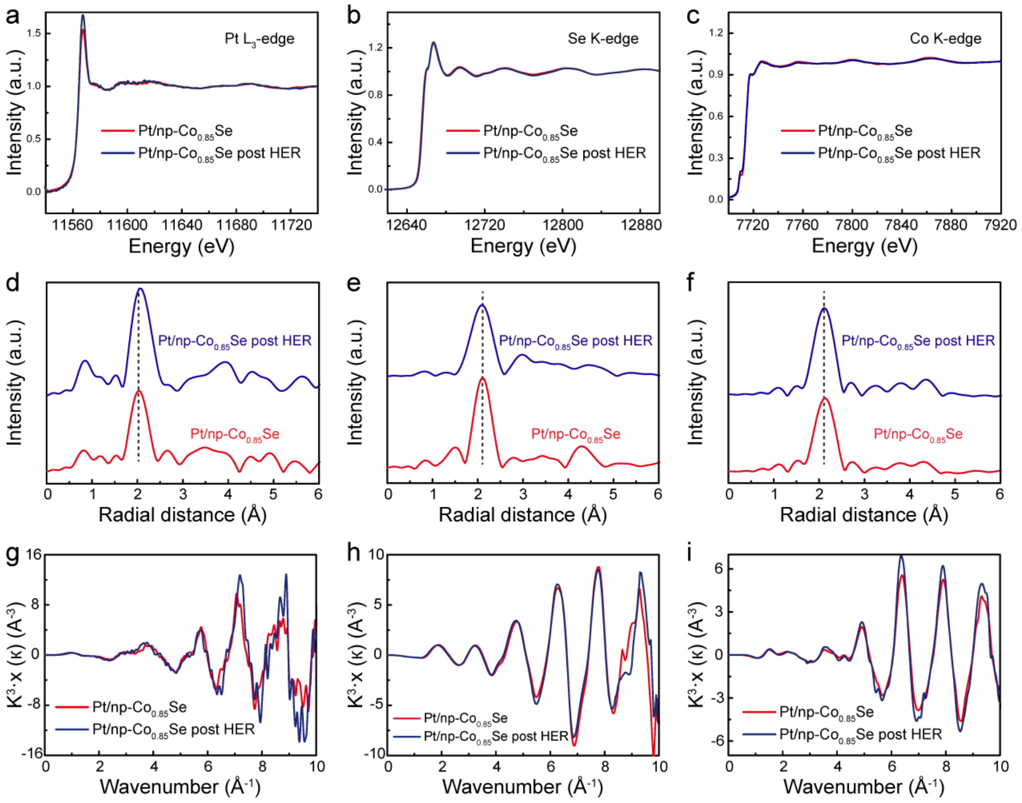

Supplementary Figure 16. XAS characterizations after long-time operation

(**a-c**) XANES spectra at Pt L_3-, Se K-, and Co K-edges. (**d-f**) FT-EXAFS spectra in real space at Pt L_3-, Se K-, and Co K-edges. (**g-i**) EXAFS $\chi(k)$ signals at Pt L_3-, Se K-, and Co K-edges.

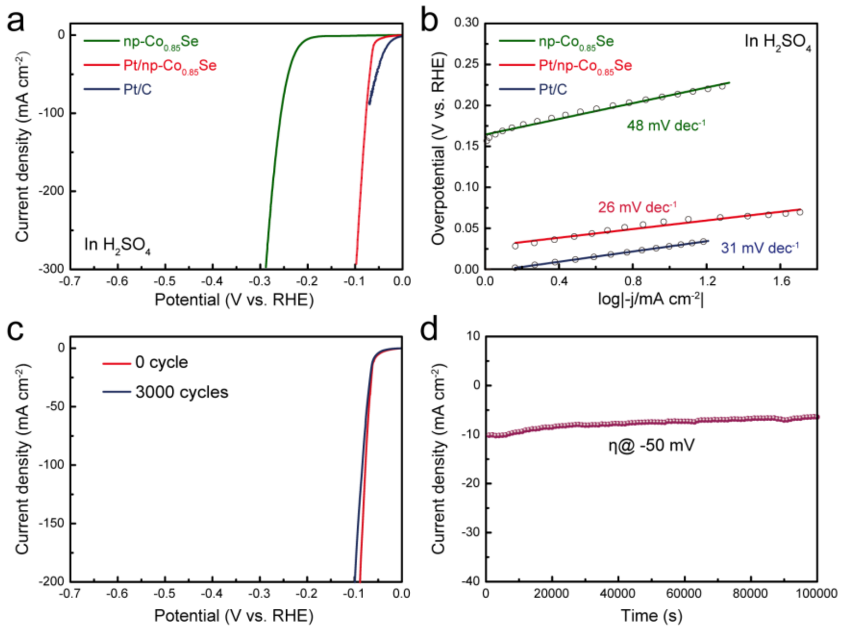

Supplementary Figure 17. HER performance in acidic electrolyte

(**a**) HER polarization curves and (**b**) tafel plots of np-$Co_{0.85}Se$, Pt/np-$Co_{0.85}Se$ and commercial Pt/C in 0.5 M H_2SO_4. (**c**) Accelerated cyclic voltammetry cycling test of Pt/np-$Co_{0.85}Se$, sweep rate: 100 mV s^{-1}. (**d**) Time-dependent current density curve at ~50 mV vs. RHE in 0.5 M H_2SO_4.

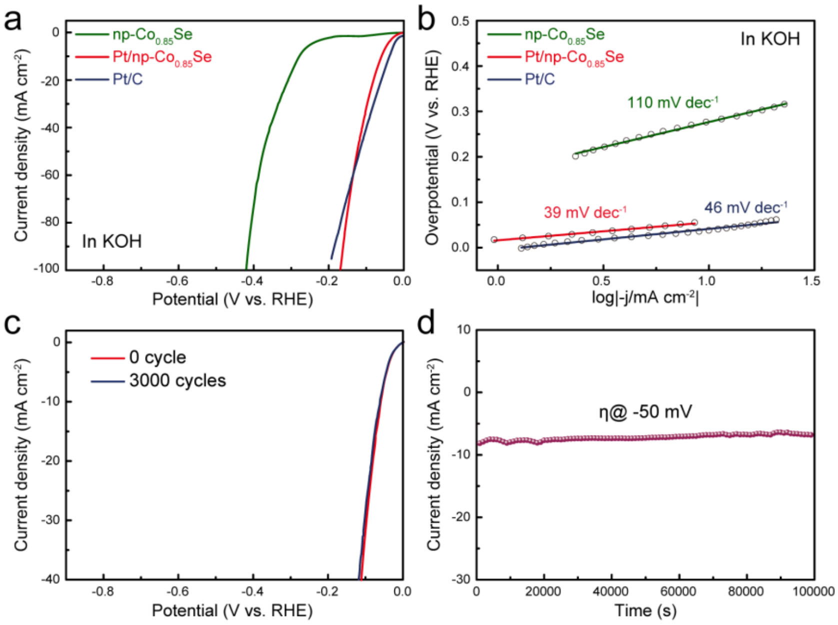

Supplementary Figure 18. HER performance in basic electrolyte

(**a**) HER polarization curves and (**b**) tafel plots of np-$Co_{0.85}Se$, Pt/np-$Co_{0.85}Se$ and commercial Pt/C in 1.0 M KOH. (**c**) Accelerated cyclic voltammetry cycling test of Pt/np-$Co_{0.85}Se$, sweep rate: 100 mV s^{-1}. (**d**) Time-dependent current density curve at ~50 mV vs. RHE in 1.0 M KOH.

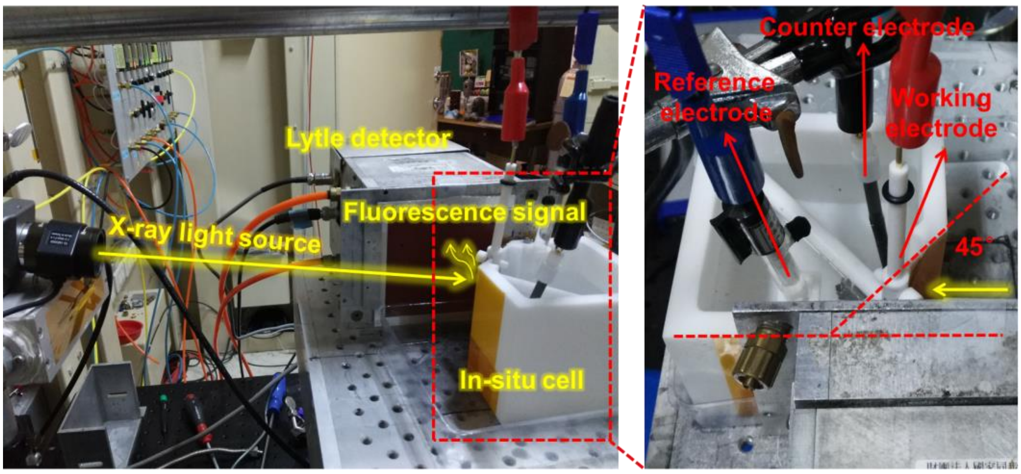

Supplementary Figure 19. The detail of the in-situ XAS measurement

For the in-situ XAS measurements, an electrochemical workstation and a custom-made poly tetra fluoroethylene cell equipped with a Pt/np-$Co_{0.85}Se$ working electrode, a carbon rod counter electrode, and a saturated calomel reference electrode were used. The window of the cell was mounted at an angle of roughly 45 ° with respect to both the incident beam and the detector. All spectra of the samples were measured in fluorescence mode.

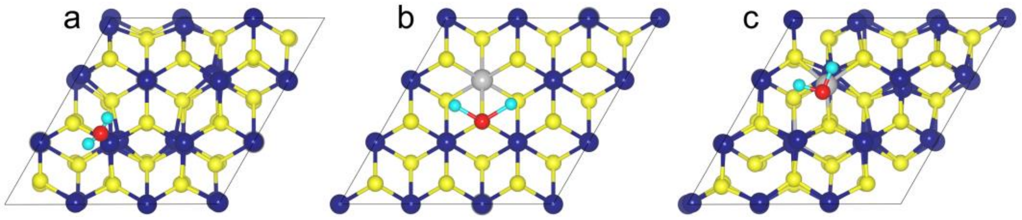

Supplementary Figure 20. H_2O adsorption on different atom sites

The adsorption sites of H_2O molecule on the top of Co (**a**), Se (**b**), and Pt (**c**) atoms in the optimized (004) facet of Pt/$Co_{0.85}$Se, respectively.

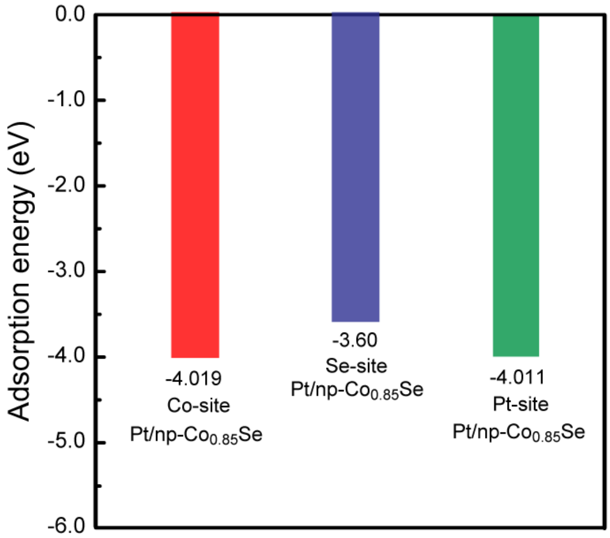

Supplementary Figure 21. Comparison of the adsorption energy of H_2O molecule on different atoms on the (004) facet of Pt/$Co_{0.85}Se$

As **Supplementary Figure 21** shown, the H_2O molecule on the Co atom sites exhibits the lowest water adsorption energy of -4.019 eV than that of Pt sites (-4.011eV) and Se sites (-3.60 eV), which indicates that the initial H_2O molecules are willing adsorbed on the Co sites of Pt/$Co_{0.85}Se$.

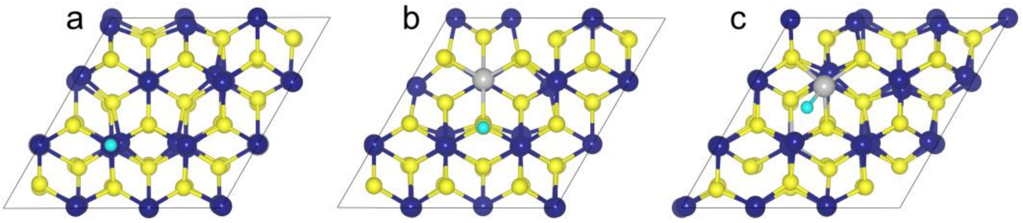

Supplementary Figure 22. H adsorption on different atom sites

The adsorption sites of H atom on the top of Co (**a**), Se (**b**) and Pt (**c**) atoms on the (004) facet of Pt/$Co_{0.85}Se$, respectively. The surface of Pt/$Co_{0.85}Se$ was built by replacing Co atoms with Pt atoms in the (004) facet of $Co_{0.85}Se$. Different Pt distributed positions in initial $Co_{0.85}Se$ have been evaluated to determine the most stable surface as structure model of the calculate hydrogen adsorption. The altered structures can modulate the surface states and charge distribution of Pt/$Co_{0.85}Se$, thus optimizing the Gibbs free-energy (ΔG_{H*}), further improving the HER.

Supplementary Tables

Supplementary Table 1. Comparison of overpotential (η) at current density of -10 mA cm^{-2}, Tafel slope and mass activity of Pt/np-$Co_{0.85}Se$ with recently reported Pt-based catalysts.

Catalysts with electrodepositing atomic scale Pt.

Catalysts	electrolyte	η_{10} (mV)	Tafel slope (mV dec^{-1})	Mass activity at η=100 mV (A mg^{-1})	Ref.
Pt/np-$Co_{0.85}Se$	1.0 M PBS	55	35	1.32	This work
Pt/np-$Co_{0.85}Se$	0.5 M H_2SO_4	58	26	13.57	This work
Pt/np-$Co_{0.85}Se$	1.0 M KOH	58	39	1.28	This work
Pt/C	1.0 M PBS	46	37	0.12	This work
Pt/C	0.5 M H_2SO_4	29	31	0.78	This work
Pt/C	1.0 M KOH	40	46	0.20	This work
#400-SWNT/Pt	0.5 M H_2SO_4	27	38	About 3.30	1
#PtSA-NT-NF	1.0 M PBS	24	30	About 0.36	2
#PtSA-NT-NF	0.5 M H_2SO_4	30	-	About 0.93	2
#PtSA-NT-NF	1.0 M KOH	20	-	About 0.54	2

#er-WS_2-Pt	0.5 M H_2SO_4	About 45	27	-	3
#er-WS_2-Pt	1.0 M KOH	About 48	65	-	3
#ep-WS_2-Pt	0.5 M H_2SO_4	About 140	50	-	3
#ep-WS_2-Pt	1.0 M KOH	About 190	124	-	3
#Pt-2H-MoS_2	0.5 M H_2SO_4	312	109	-	4
#Pt-1T-MoS_2	0.5 M H_2SO_4	210	104	-	4
Pt@PCM	0.5 M H_2SO_4	105	65.3	About 0.10	5
Pt@PCM	1.0 M KOH	139	73.6	-	5
Pt@MoS_2/NiS_2	0.5 M H_2SO_4	34	41	About 7.30	6
Pt–MoS_2	0.5 M H_2SO_4	53	40	About 1.74	7
Pt-$Co(OH)_2$/CC	1.0 M PBS	84	-	About 0.03	8

Supplementary Table 2. Comparison of onset potential, overpotential (η) at current density of -10 mA cm^{-2} and Tafel slope of Pt/np-$Co_{0.85}Se$ with recently reported catalysts in neutral electrolyte.

Catalysts	electrolyte	Onset potential @ j = -1 mA cm^{-1} (mV vs. RHE)	η_{10} (mV)	Tafel slope (mV dec^{-1})	Ref.
Pt/np-$Co_{0.85}Se$	1.0 M PBS	12	55	35	This work
np-$Co_{0.85}Se$	1.0 M PBS	75	201	90	This work
Pt/C	1.0 M PBS	15	46	37	This work
Co_9S_8/NC@MoS_2	1.0 M PBS	45	261	126.1	9
$Ni_{1-x}Co_xSe_2$	1.0 M PBS	-	82	78	10
CoP/CC	1.0 M PBS	65 (j=2 mA cm^{-1})	-	93	11
Co-HNP	1.0 M PBS	27 (j=2 mA cm^{-1})	87	41.7	12
$NiCo_2Px$	1.0 M PBS	-	63	63.3	13
CoS/FTO	1.0 M PBS	87 (j=2 mA cm^{-1})	170	93	14
SiO_2/PPy NTs-CFs	1.0 M PBS	-	70	100	15
Co/CoP	1.0 M PBS	-	138	72	16
CoO/$CoSe_2$	0.5 M PBS	200 (j=2 mA cm^{-1})	337	131	17
FeP/CC	1.0 M PBS	-	115	70	18

CoP/Ti	0.2 M PBS	102 (j=2 mA cm^{-1})	149	58	19
CoS_2/Ti	1.0 M PBS	-	260	129	20
Co-SNP/CC	1.0 M PBS	29.5 (j=2 mA cm^{-1})	111	74	12

Supplementary Table 3. Comparison of the TOF value of Pt/np-$Co_{0.85}Se$ with other reported catalysts.

Catalysts	electrolyte	TOF at η=100 mV	Ref.
Pt_{5000}/np-$Co_{0.85}Se$	1.0 M PBS	3.93	This work
np-$Co_{0.85}Se$	1.0 M PBS	0.17	This work
CoP_X	1.0 M KOH	0.015	13
CoP	0.5 M H_2SO_4	0.046	21
Co-NG	0.5 M H_2SO_4	0.101	22
CoN_X/C	0.5 M H_2SO_4	0.39	23
$NiCo_2Px$	1.0 M KOH	0.056	13
Pt/C	0.5 M H_2SO_4	1.25	24
Ni/GD	0.5 M H_2SO_4	1.59	25
PtRu/RFCS	0.5 M H_2SO_4	0.375	24
CoP/CC	1.0 M PBS	0.725 (75 mV)	21
p-1T-MoS_2	0.5 M H_2SO_4	0.5 (153 mV)	26
Pt@PCM	0.5 M H_2SO_4	10 (200 mV)	5

Supplementary Notes

Supplementary Note 1: Mechanism of Co vacancies formation and Pt species reduction.

Co vacancies formation. Nanoporous $Co_{0.85}Se$ fabricated by electrochemically selective etching possesses a self-supporting three-dimensional nanoarchitecture. The low-coordinated Co atoms on the surface of $Co_{0.85}Se$ ligaments (**Ref. 27**) are prone to be dissolved (chemical dissolution or electrochemical dissolution) in acidic solution to form Co vacancies during the cathodic scan. The Se K-edge EXAFS were used to confirm the formation of Co vacancies. **Supplementary Figure 6** shows that, after 5000 potential cycles using graphite sheet as counter electrode in a three-electrode cell containing 0.5 M H_2SO_4, the Se-Co shell scattering was decreased, indicating the loss of Co atoms, thus providing anchor sites for Pt atoms.

Pt species reduction. During the anodic scan, Pt is dissolved via chemically/electrochemically approach. The oxidation of Pt attributed to the adsorption of oxygenated species can be described by equation 1 and 2, where the oxidation species determined by the applied anodic potential (**Ref. 28**).

$$Pt + H_2O \rightarrow PtO + 2H^+ + 2e^- \qquad (1)$$

$$PtO + H_2O \rightarrow PtO_2 + 2H^+ + 2e^- \qquad (2)$$

The exposure of oxidized Pt atoms at the surface creates conditions for its chemical dissolution during anodic scan (Equation 3 & 4). Furthermore, Pt-O can be reduced, resulting in low-coordinated Pt which is much easier to dissolve than chemical dissolution during anodic scan (electrochemical dissolution, Equation 5).

$Pt\text{-}O + 2H^+ \rightarrow Pt^{2+} + H_2O$ (3)

$Pt\text{-}O_2 + 4H^+ \rightarrow Pt^{4+} + 2H_2O$ (4)

$Pt \rightarrow Pt^{2+} + 2e^-$ (5)

Finally, Pt species dissolved from the Pt foil will to be slowly reduced and trapped by Co vacancies, forming Pt/np-$Co_{0.85}$Se.

Supplementary Note 2: Calculation of the Pt mass activity.

In order to compare the Pt mass activities of Pt/C and Pt/np-$Co_{0.85}$Se, their activity values have been normalized to Pt loadings. For Pt/np-$Co_{0.85}$Se, the contribution of np-$Co_{0.85}$Se has been deducted. The overpotential of -100 mV was selected to evaluate the mass activity. The details are as follows:

$$j_{mass}^{\mathrm{Pt/np-Co_{0.85}Se}} = \frac{j_{area}^{Pt/np-Co_{0.85}Se} - j_{area}^{np-Co_{0.85}Se}\ (mA\ cm^{-2})}{mass_{Pt}} = \frac{35.449 - 1.325\ (mA\ cm^{-2})}{0.0258\ (mg\ cm^{-2})}$$

$$= 1.323\ A\ mg^{-1} \qquad (6)$$

$$j_{mass}^{Pt/C} = \frac{j_{area}^{Pt/C}}{mass_{Pt}} = \frac{24.630\ (mA\ cm^{-2})}{0.2040\ (mg\ cm^{-2})} = 0.121\ A\ mg^{-1} \qquad (7)$$

Supplementary Note 3: Calculation of the active site density and TOF.

TOF is the best figure of merit to compare the intrinsic activities of catalysts with different loadings or surface areas. In order to calculate the active surface site density and per-site TOF of catalyst, we adopt the method applied by **Ref. 25**. The per-site TOF values can be calculated according to the following equation:

$$TOF = \frac{Total\ number\ of\ hydrogen\ turnover/geometric\ area\ (cm^{-2})}{Number\ of\ active\ sites/geometric\ area\ (cm^{-2})} \qquad (8)$$

The number of total hydrogen turnovers is calculated from the current density according to:

Number of hydrogens

$$= \left(j\,\frac{mA}{cm^2}\right)\left(\frac{1\,C\,s^{-1}}{1000\,mA}\right)\left(\frac{1\,mol\,e^{-1}}{96485.3\,C}\right)\left(\frac{1\,mol\,H_2}{2\,mol\,e^{-}}\right)\left(\frac{6.022\times10^{23} molecules\,H_2}{1\,mol\,H_2}\right)$$

$$= 3.12\times10^{15}\,H_2\,s^{-1}cm^{-2}\,per\,mA\,cm^{-2} \qquad (9)$$

The loading of Pt was determined from the ICP-OES measurement (1.03 wt%).

The active site density of Pt is (**Ref. 29**):

$$\left(0.15\,mg\times\frac{1.032}{100}\right)\times\left(\frac{1\,mmol}{195.078\,mg}\right)\times6.022\times10^{20}\,sites\,mmol^{-1}\times\frac{1}{0.15\times223.44}\,cm^{-2}$$

$$= 1.426\times10^{14}\,Pt-sites\,cm^{-2} \qquad (10)$$

The active site densities of Co and Se are estimated by using the method suggested by **Ref. 30**:

$$Pt/np-Co_{0.85}Se\text{: } 9.021\times10^{14}\,atoms\,cm_{real}^{-2}\times(BET_1)\times(mass)$$

$$= 3.032\times10^{16}\,Co\,and\,Se-sites\,cm^{-2} \qquad (11)$$

$$np-Co_{0.85}Se\text{: } 9.021\times10^{14}\,atoms\,cm_{real}^{-2}\times(BET_2)\times(mass)$$

$$= 2.406\times10^{16}\,Co\,and\,Se-sites\,cm^{-2} \qquad (12)$$

Finally, the current densities from the polarization curves can be converted into TOF values according to:

$$TOF_{Pt/np-Co_{0.85}Se} = \frac{3.12\times10^{15}\,H_2\,s^{-1}\,cm^{-2}\,per\,mA\,cm^{-2}}{1.426\times10^{14}\,Pt-sites\,cm^{-2}+3.032\times10^{16}\,Co\,and\,Se-sites\,cm^{-2}}\times j$$

$$= 0.103\,j \qquad (13)$$

$$TOF_{np-Co_{0.85}Se} = \frac{3.12\times10^{15}\,H_2\,s^{-1}\,cm^{-2}\,per\,mA\,cm^{-2}}{2.406\times10^{16}\,Co\,and\,Se-sites\,cm^{-2}}\times j = 0.129\,j \qquad (14)$$

Therefore, the TOF values of np-$Co_{0.85}Se$ and Pt/np-$Co_{0.85}Se$ catalysts were calculated to be: TOF (-0.1 V vs. RHE) = 0.17 H_2 s^{-1} and 3.93 H_2 s^{-1}, respectively.

Note that because the nature of the active sites and the real surface area of the np-$Co_{0.85}Se$ and Pt/np-$Co_{0.85}Se$ catalysts are difficult to determine accurately, the

results provided here are just indirect estimation.

Supplementary Note 4: Ex-situ XAS and In-situ XAS measurements.

The ex-situ X-ray absorption spectroscopy was performed at the beamline BL01C1 at National Synchrotron Radiation Research Center (NSRRC, Taiwan). The Pt-L_3, Co-K, and Se K-edges XAS spectra of Pt/np-$Co_{0.85}Se$ and the Co-K and Se K-edges XAS spectra of np-$Co_{0.85}Se$ were performed in the fluorescence mode using a Lytle detector. To optimize the XAS measurements, several XAS spectra at different positions on each sample were collected. No obviously difference was found among these XAS spectra due to the uniformity of the sample. In these conventional fluorescence detection measurements, the background from elastic and Compton scattering was reduced using a combination of Z-1 filters with Soller slits.

The in-situ XANES and EXAFS data were obtained on beamline BL01C1 at NSRRC in the fluorescence mode using a Lytle detector with a step-size of 0.25 eV at room temperature. For the in-situ XAS measurements, an electrochemical workstation (Ivium CompactStat.h) and a custom-made poly tetra fluoroethylene (PTFE) cell were used. The catalysts were coated on the carbon cloth via drop casting to form a working electrode. Then, the PTFE cell was equipped with a Pt/np-$Co_{0.85}Se$ working electrode, a carbon rod counter electrode, and a saturated calomel reference electrode in 1.0 M PBS. Finally, the window of the PTFE cells was mounted at an angle of roughly 45° with respect to both the incident beam and the detector (**Supplementary Fig. 19**). During the measurements, the different potentials of OCV, -0.1, and -0.2 V versus RHE were applied to the system.

Supplementary Note 5: Theoretical calculations.

DFT calculations were performed by using the Vienna Ab-initio Simulation Package (VASP). The projected augmented wave (PAW) potential and generalized gradient approximation of the Perdew-Burke-Ernzerhof functional were employed to describe the electron-ion interaction and exchange-correlation energy, respectively. The DFT-D3 empirical correction method was employed to accurately describe the van der Waals interactions. The energy cutoff for the plane-wave expansion was set to 400 eV. The energy convergence was set to 10^{-5} eV, and the residual force on each atom was smaller than 0.01 eV/Å for structural relaxations. To investigate the adsorption of isolated Pt atoms on the surface of np-$Co_{0.85}Se$, we calculated spin density distribution in the 3×3×1 $Co_{0.85}Se$ supercell doped with two Pt atoms.

Supplementary References

1. Tavakkoli, M. et al. Electrochemical Activation of Single-Walled Carbon Nanotubes with Pseudo-Atomic-Scale Platinum for the Hydrogen Evolution Reaction. *ACS Catal.* **7**, 3121-3130 (2017).

2. Zhang, L. et al. Potential-Cycling Synthesis of Single Platinum Atoms for Efficient Hydrogen Evolution in Neutral Media. *Angew. Chem. Int. Ed.* **56**, 13694-13698 2017).

3. Tang, K. et al. High Edge Selectivity of In Situ Electrochemical Pt Deposition on Edge-Rich Layered WS_2 Nanosheets. *Adv. Mater.* **30**, 1704779 (2018).

4. Xuan, N. et al. Single-Atom Electroplating on Two Dimensional Materials. *Chem. Mater.* **31**, 429-435(2019).

5. Zhang, H. et al. Dynamic traction of lattice-confined platinum atoms into mesoporous carbon matrix for hydrogen evolution reaction. *Sci. Adv.* **4**, eaao6657 (2018).

6. Guan, Y. et al. Ganoderma-Like MoS_2 /NiS_2 with Single Platinum Atoms Doping as an Efficient and Stable Hydrogen Evolution Reaction Catalyst. *Small* **14**, 1800697 (2018).

7. Huang, X. et al. Solution-phase epitaxial growth of noble metal nanostructures on dispersible single-layer molybdenum disulfide nanosheets. *Nat. Commun.* **4**, 1444 (2013).

8. Xing, Z. et al. Ultrafine Pt Nanoparticle-Decorated $Co(OH)_2$ Nanosheet Arrays with Enhanced Catalytic Activity toward Hydrogen Evolution. *ACS Catal.* **7**, 7131-7135 (2017).

9. Li, H. et al. Hierarchical Porous Co_9S_8/Nitrogen-Doped Carbon@MoS_2 Polyhedrons as pH Universal Electrocatalysts for Highly Efficient Hydrogen Evolution Reaction. *ACS Appl. Mater. Interfaces* **9**, 28394-28405 (2017).

10. Liu, B. et al. Nickel-Cobalt Diselenide 3D Mesoporous Nanosheet Networks Supported on Ni Foam: An All-pH Highly Efficient Integrated Electrocatalyst for Hydrogen Evolution. *Adv. Mater.* **29**, 606521 (2017).

11. Tian, J. et al. Self-supported nanoporous cobalt phosphide nanowire arrays: an efficient 3D hydrogen-evolving cathode over the wide range of pH 0-14. *J. Am. Chem. Soc.* **136**, 7587-7590 (2014).

12. Liu, B, et al. Cobalt-Nanocrystal-Assembled Hollow Nanoparticles for Electrocatalytic Hydrogen Generation from Neutral-pH Water. *Angew. Chem. Int. Ed.* **55**, 6725-6729 (2016).

13. Zhang, R. et al. Ternary $NiCo_2Px$ Nanowires as pH-Universal Electrocatalysts for Highly Efficient Hydrogen Evolution Reaction. *Adv. Mater.* **29**, 1605502 (2017).

14. Sun, Y. et al. Electrodeposited cobalt-sulfide catalyst for electrochemical and photoelectrochemical hydrogen generation from water. *J. Am. Chem. Soc.* **135**, 17699-17702 (2013).

15. Feng, J. X. et al. Silica-Polypyrrole Hybrids as High-Performance Metal-Free Electrocatalysts for the Hydrogen Evolution Reaction in Neutral Media. *Angew. Chem. Int. Ed.* **56**, 8120-8124 (2017).

16. Xue, Z.-H. et al. Janus Co/CoP Nanoparticles as Efficient Mott-Schottky Electrocatalysts for Overall Water Splitting in Wide pH Range. *Adv. Energy Mater.* **7**,

1602355 (2017).

17. Li, K. et al. Anchoring CoO Domains on $CoSe_2$ Nanobelts as Bifunctional Electrocatalysts for Overall Water Splitting in Neutral Media. *Adv. Sci.* **3**, 1500426 (2016).

18. Tian, J. et al. FeP nanoparticles film grown on carbon cloth: an ultrahighly active 3D hydrogen evolution cathode in both acidic and neutral solutions. *ACS Appl. Mater. Interfaces* **6**, 20579-20584 (2014).

19. Pu, Z. et al. CoP Nanosheet Arrays Supported on a Ti Plate: An Efficient Cathode for Electrochemical Hydrogen Evolution. *Chem. Mater.* **26**, 4326-4329 (2014).

20. Zhang, H. et al. Highly Crystallized Cubic Cattierite CoS_2 for Electrochemically Hydrogen Evolution over Wide pH Range from 0 to 14. *Electrochim. Acta* **148**, 170-174 (2014).

21. Popczun, E. J. et al. Highly active electrocatalysis of the hydrogen evolution reaction by cobalt phosphide nanoparticles. *Angew. Chem. Int. Ed.* **53**, 5427-5430 (2014).

22. Fei, H. et al. Atomic cobalt on nitrogen-doped graphene for hydrogen generation. *Nat. Commun.* **6**, 8668 (2016).

23. Liang, H. W. et al. Molecular metal-Nx centres in porous carbon for electrocatalytic hydrogen evolution. *Nat. Commun.* **6**, 7992 (2015).

24. Li, K. et al. Enhanced electrocatalytic performance for the hydrogen evolution reaction through surface enrichment of platinum nanoclusters alloying with ruthenium in situ embedded in carbon. *Energy Environ. Sci.* **11**, 1232-1239 (2018).

25. Xue, Y. et al. Anchoring zero valence single atoms of nickel and iron on graphdiyne for hydrogen evolution. *Nat. Commun.* **9**, 1460 (2018).

26. Yin, Y. et al. Contributions of Phase, Sulfur Vacancies, and Edges to the Hydrogen Evolution Reaction Catalytic Activity of Porous Molybdenum Disulfide Nanosheets. *J. Am. Chem. Soc.* **138**, 7965-7972 (2016).

27. Fujita, T. et al. Atomic origins of the high catalytic activity of nanoporous gold. *Nat. Mater.* **11**, 775-780 (2012).

28. Chen, R. et al. Use of Platinum as the Counter Electrode to Study the Activity of Nonprecious Metal Catalysts for the Hydrogen Evolution Reaction. *ACS Energy Lett.* **2**, 1070-1075 (2017).

29.Tiwari, J. N. et al. Multicomponent electrocatalyst with ultralow Pt loading and high hydrogen evolution activity. *Nat. Energy* **3**, 773-782 (2018).

30. Kibsgaard, J. et al. Designing an improved transition metal phosphide catalyst for hydrogen evolution using experimental and theoretical trends. *Energ. Environ. Sci.* **8**, 3022-3029 (2015).
